# Effect of Low Power Laser on Incisional Wound Healing

**Published:** 2006-07-01

**Authors:** Masoud Parirokh, Shahriar Dabiri, AliReza Bahrampour, Mahmoud Homayon Zadeh, Mohammad Jafar Eghbal

**Affiliations:** 1 *Department of Endodontics, Dental School, Kerman University of Medical Sciences, Kerman, Iran*; 2 *Department of Pathology, Medical School, Kerman University of Medical Sciences, Kerman, Iran*; 3 *Department of Mathematics, Vali-Asr Rafsanjan University of Medical Sciences, Rafsanjan, Iran*; 4 *General Practitioner, Rafsanjan, Iran*; 5 *Department of Endodontics, Dental Research Center, Shahid Beheshti University of Medical Sciences, Tehran, Iran*

**Keywords:** He-Ne Laser, Healing, Incisional Wound, Low Power Laser

## Abstract

**INTRODUCTION:** The effectiveness of low power lasers for incisional wound healing, because of conflicting results of previous research studies, is uncertain. Therefore, this study was carried out to evaluate low power laser effects on incisional wound healing.

**MATERIALS AND METHODS:** Incisional wound was produced on thirty-six mature male guinea pigs under general and local anesthesia. In half of the cases, He-Ne laser radiations were used for five minutes and the rest were left untreated. Animals were divided into six groups of six animals each that were killed after 3, 5 and 14 days. After histopathology processing and H&E staining, specimens were examined for acute and chronic inflammations, epithelial cell migration, epithelial seal and barrier formation, fibroblast migration, fibrosis, clot formation and granulation tissue formation. Mann-Whitney U and the Wilcoxon tests were used for statistical analysis.

**RESULTS:** Statistically significant differences were found between fibroblast migration, acute and chronic inflammation of radiated groups and the control group at *5 *days interval (p<0.05). There was no statistically significant difference at 3 and 14 days between laser radiated and control groups.

**CONCLUSION:** This study showed that He-Ne laser had beneficial effects on incisional wound healing particularly at 5 days interval; however, further research on chronic ulcers is recommended.

## INTRODUCTION

LASER is an acronym for light amplification by stimulated emission of radiation and has been used in medical sciences since 1960s. Laser in dentistry is often associated with high power lasers that bum or disintegrate tissues (1,2). Little has been published about the use of low power lasers in dental practice.

Initially, Mester *et al. *published a report on the beneficial effect of this type of laser (3).

Low power lasers do not affect tissue thermally but act to increase the rate of repair of injured tissue (4). Studies have shown that low power lasers can affect the biological functions of macrophages (5), angiogenesis (6) and Low power lasers such as Helium-Neon (He-Ne), Ruby, Gallium-Aluminum-Asnium (Ga-Al-As) has been reported to have beneficial effects on tissue wound healing in animals as well as in human tissue culture (7,8).

Laser therapy could be useful as a treatment modality in myofascial pain syndrome because of its noninvasiveness, ease, and short-term application (9). Also it was reported to reduce post extraction pain and swelling and to increase rates of wound healing (2). However, some studies in which red spectrum laser were used resulted in confusing data and conflicting findings. Some of these studies indicated that the biostimulation effect did not occur in all but some cases of laser irradiation (2,7,8,10). Few controlled studies were carried out in order to identify the beneficial effects of He­ Ne laser bio-stimulation. Ethical concern, bulky equipment and difficulties with sound study design have precluded a precise evaluation of laser bio-stimulation (11). Most of earlier studies on oral tissues were observational (12), or clinical data collection on pain, swelling and discomfort (2,13,14). Therefore, the purpose of this study was to determine the histopathological effect of the He-Ne laser on oral surgical wound healing.

## RESULTS

Two laser and one control specimens at 3 days interval were excluded because of processing problem. Histopathologic results of the remaining specimens were as follows:

3-Days interval: All specimens in both control and laser irradiated groups showed epithelial migration and crust between two edges of surgical incision area. Epithelial seal could be observed in one of the laser radiated (LR) specimens. In the rest of specimens the epithelial seal and barrier did not form. Polymorphonuclears, macrophages and plasma cells were observed in both control and experimental groups with no significant differences.

5-Days interval: Significant differences were observed between 3 and 5 days in both LR and control groups. Healing in all 5^th ^day specimens was better than 3^rd^ day animals.

There was significant differences between LR and control group in relation to the fibroblast migration, acute and chronic inflammation, clot formation and fibrosis (p<0.05) (Table 1).

**Figure 1:**
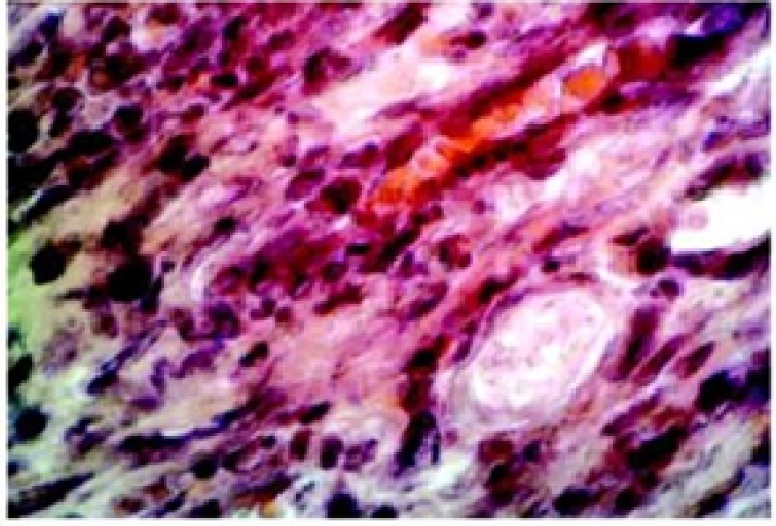
Inflammatory cells in 5-day non radiated group showing smaller fibroblast and more inflammatory cells (×20)

**Figure 2:**
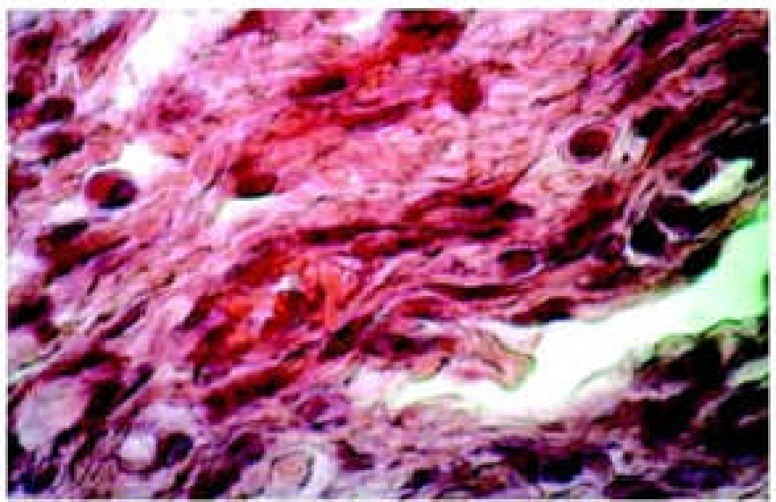
Five-day radiated group showing decreasing number of inflammatory cells and enlarged fibroblasts compared with smaller size of non-radiated group (×20)

**Figure 3:**
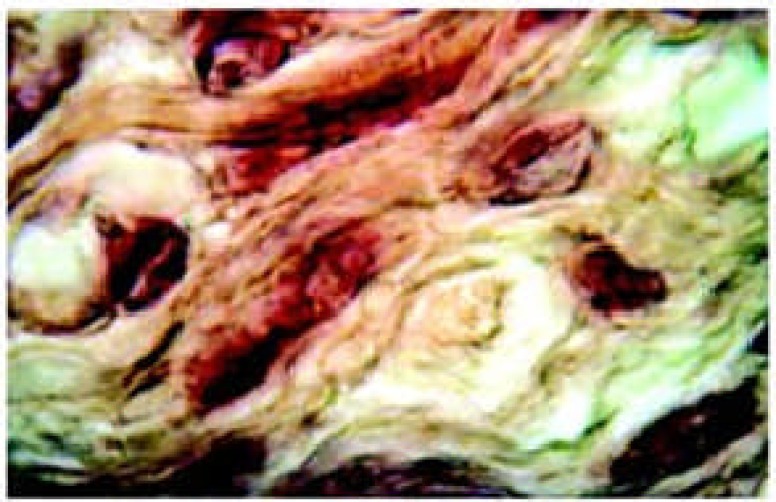
Higher magnification of plumped fibroblast (×40)

The number of inflammatory cells in LR group was lower than the control group (Figure 1), (Figure 2). Plumped fibroblasts (Figure 3) were very evident in the LR specimens (p<0.05).

Although specimens of control group showed more tissue maturation than radiated group, there were no statistical differences between epithelial seal and barrier formation between LR and control groups (p>0.05).

14-Days interval: There was no significant difference between LR and control groups at 14 days. Epithelial barrier was completed and inflammation and fibrosis were similar in both groups.

## DISCUSSION

Promotion of healing is of paramount importance in medicine, particularly in diabetic and immuno-compromised patients (16). There have been various studies performed on low power laser; however, conflicting results and few oral researches motivated the researchers to conduct this study. In this study, He-Ne laser was used and the results showed that in the LR group particularly at *5 *days interval, healing was more evident than non radiated group. This was similar to the results of some previous studies (13,17-19) although it was in conflict with the results of many other investigations (8,10,11,14). Researchers of previous studies believed that the differences between fluency­ energy level in tissues (2,4,13), frequency of radiation (12), systemic effect (17,20,21) and the type of ulcer (19) would influence the results of low power laser exposures and produced conflicting results. It is believed that the optimum tissue-healing rates at He-Ne laser exposure levels exist between I J/Cm2 -20J/Cm^2^ (2). This amount of energy could induce metabolic changes within the cells. In this study, the energy level produced in tissue was 2.5 J/cm^2^**.** Results showed that fibroblast proliferation was significantly more evident in the LR than control group in 5 days interval which was in agreement with previous studies in which low power laser beneficial effects were demonstrated (2,18,19).

**Table 1:** Statistical analysis of laser radiated and control group at 5 days interval

**Time intervals**	Mean Rank								
	CT	AI	CI	EM	ES	EB	FM	GT	FR
**LR Group**	6.5	9.5	8.5	5.75	5.25	5.67	8.5	8	6
**Control Group**	1.5	3.5	4.5	7.25	7.75	7.33	4.5	5	7
**Significancy**	0.00	0.002	0.03	0.386	0.176	0.34	0.019	0.058	0.0317

*LR: Laser radiated, CT: clot, AI: Acute Inflammation, CI: Chronic Inflammation, EM: Epithelial Migration, ES: Epithelial Seal, EB: Epithelial Barrie, FM: Fibroblast Migration, GT: Granulation Tissue formation, FR: Fibrosis*

Studies of Mester *et al. *and Abergel *et al. *showed that the frequency of radiation could improve tissue healing rates (3,18). However, in this study, despite a single radiation exposure significant differences were found between LR and control groups in inflammation and fibroblast migration at 5-day intervals. Neiburger and Yu *et al. *showed the same finding after single laser radiation (2,4). Funk *et al.* showed those 30 minutes after laser radiation of peripheral mononuclear blood cells, IL1α, lL2, TNFα and INFγ increased significantly (7). It might be one of the reasons that even with one radiation exposure, the beneficial effects of He-Ne laser could be observed in the present study.

The systemic effect of these cytokines was confirmed by Belkin and Schwartz as well as Karu and Inoue (17,19,20). Therefore, in many studies, as both laser radiation and control procedure were performed on the same patient, the laser radiation would not produce precise results (8,14,21). This is the reason for using different guinea pigs for control and LR groups in the present investigation.

The type of ulcer could affect radiation response. Many researchers believed that old ulcers, because of low oxygen concentration, PH and nutrients showed a better response to low power laser than fresh ulcer (19). This study, as well as others, demonstrated that He-Ne laser has beneficial effects on fresh ulcers (2,18,22,23).

## CONCLUSION

In conclusion this study showed that He­Ne laser has had beneficial effects on incisional wound healing particularly at 5 days interval. However, further research on chronic ulcers is recommended.

## References

[1] Kimura Y, Wilder-Smith P, Matsumoto K (2000). Lasers in endodontics. Int Endod J.

[2] Neiburger EJ (1997). Accelerated healing of gingival incisions by the He-Ne diode laser: A preliminary study. Gen Dent.

[3] Mester E, Spiry T, Szende B, Tota JG (1971). Effect of laser rays on wound healing. Am J Surg.

[4] Yu HS, Chang KL, YU CL, Chen JW, Chen GS (1996). Low-energy He-Ne laser irradiation stimulates. J Invest Dermatol.

[5] Dube A, Bansal H, Gupta PK (2003). Modulation of macrophage structure and function by low level He-Ne laser irradiation. Photochem Photobiol Sci.

[6] Garavello I, Baranauskas V, da Cruz-Hofling MA (2004). The effects of low laser irradiation on angiogenesis in injured rat tibiae. Histol Histopathol.

[7] Funk JO, Kruse A, Kichner H (1992). Cytokine production after He-Ne laser irradiation in cultures of human peripheral blood mononuclear. J Photochem Photobiol b: Biol.

[8] Anneroth G, Hall G, Ryden H, Zettervist L (1988). The effect of low-energy infra-red laser radiation on wound healing. Brith Assoc Oral and Maxilla Surg.

[9] Ilbuldu E, Cakmak A, Disci R, Aydin R (2004). Comparison of laser, dry needling, and placebo laser treatments in myofascial pain syndrome. Photomed Laser Surg.

[10] Colver GB, Priestley GC (1989). Failure of a He­ Ne laser to affect components of wound healing in vitro. Brith J Derm.

[11] Strange R, Moseley H, Carmaicheal A (1988). Soft lasers-have they place in dentistry?. Brith Dent J.

[12] In de Braekt MMH, Van Alphen FAM, Kuijpers-Jagtman AM, Maltha JC (1991). Effect of low level laser therapy on wound healing after palatal surgery in beagle dogs. Lasers Surg Med.

[13] Saperia D, Glassbery E, Lyons RF, Abergel RP, Baneux P, Castel JC, Dwyer RM, Uitto J (1986). Demonstration of elevated type I and type III procollagen mRNA levels in cutaneous wounds treated with He-Ne laser. Bioche Biophysics Research Commun.

[14] Masse JF, Landry RG, Rochette C, Dufour L, Morency R, D'Aoust P (1993). Effectiveness of soft laser treatment in periodontal surgery. Int Dent J.

[15] Harrison JW, Jurosky KA (1991). Wound healing in the tissues of the periodontium following periradicular surgery II: The dissectional wound. J Endod.

[16] Schindl A, Schindl M, Pemerstorfer-schon H (1999). Systemic effects of low intensity laser irradiation: results in patients with diabetic microangiopathy and literature review. American Society for Laser Medicine and Surgery (abstracts) printed in Laser Surg Med.

[17] Belkin M, Schwartz M (1989). Photochemical effects upon the cornea, skin and other tissues. Health physics.

[18] Abergel RP, Meeker CA, Lam TS, Dwyer RM, Lesavoy MA, Uitto (1984). Control of connective tissue metabolism by lasers: recent developments and future prospects. J Am Acad Derm.

[19] Karu T (1989). Photobiology of low-power laser effects. Health Physics.

[20] Inoue K, Nishiaka J, Hukuda S (1989). Suppressed tuberculin reaction in guinea pigs following laser irradiation. Laser Surg Med.

[21] Wilder-Smith P (1988). The soft laser therapeutic tool or popular placebo?. Oral Surg.

[22] Schenek P, Poreder H, Zenter K (1986). Helium­ neon laser effect auf haut undorale chleimhautgewebe. Laryng Rhinolotol.

[23] Tsuchida T, Aizawa K, Baba J, Furukawa K, Yamamoto H, Kawate N, Konaka C, Kato H, Hayata Y, Ishitsuki M (1991). Wound healing in mice using He-Ne scanning laser. J Clin Med & Surg.

